# A combinatorial CRISPR-Cas12a attack on HIV DNA

**DOI:** 10.1016/j.omtm.2022.02.010

**Published:** 2022-02-26

**Authors:** Minghui Fan, Ben Berkhout, Elena Herrera-Carrillo

**Affiliations:** 1 Laboratory of Experimental Virology, Department of Medical Microbiology, Amsterdam UMC, Academic Medical Center, University of Amsterdam, Amsterdam, the Netherlands

**Keywords:** HIV-1, CRISPR-Cas12a, lentiviral vector, dual crRNAs, cure

## Abstract

CRISPR-Cas12a is an alternative class 2 gene editing tool that may cause less off-target effects than the original Cas9 system. We have previously demonstrated that Cas12a attack with a single CRISPR RNA (crRNA) can neutralize all infectious HIV in an infected T cell line in cell culture. However, we demonstrated that HIV escapes from most crRNAs by acquisition of a mutation in the crRNA target sequence, thus providing resistance against Cas12a attack. Here, we tested the antiviral activity of seven dual crRNA combinations and analyzed the HIV proviral genomes for mutations at the target sites. We demonstrated that dual crRNA combinations exhibit more robust antiviral activity than a single crRNA attack and, more important, that the dual-crRNA therapy can prevent virus escape in long-term cultures. We confirmed the absence of any replication-competent virus in these apparently cured cultures. Surprisingly, we did not detect excision of the HIV sequences located between two Cas12a cleavage sites. Instead, we observed almost exclusively HIV inactivation by “hypermutation,” that is, the introduction of indel mutations at both target sites due to the error-prone cellular DNA repair machinery.

## Graphical abstract

**Figure:**
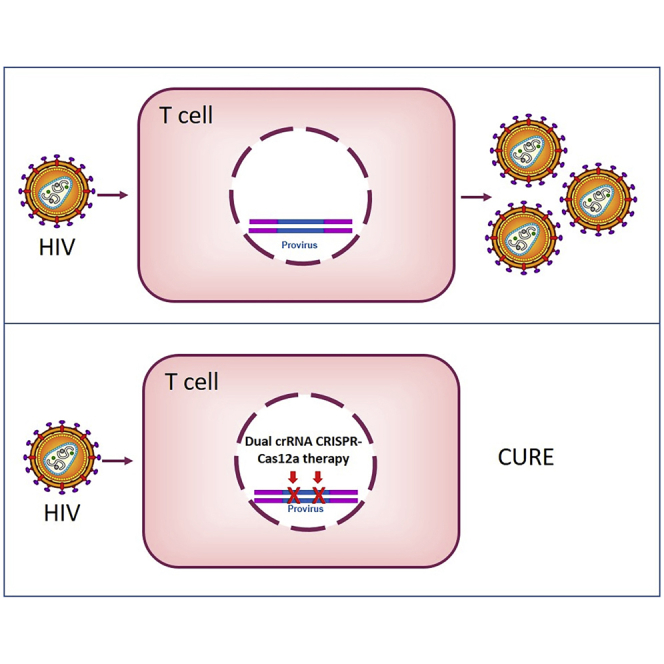

## Teaser

CRISPR-Cas12a may cause less off-target effects than the original Cas9 system. The anti-HIV activity of dual-crRNA CRISPR-Cas12a combinations was tested. The dual-crRNA therapy prevents virus escape in long-term cultures. The absence of any replication-competent virus in these apparently cured cultures was confirmed. We observed almost exclusively HIV inactivation by “hypermutation” at both target sites.

## Introduction

In the past decades, scientists have made great strides in developing HIV treatment and prevention methods, but so far, there is still no cure for AIDS. Combination antiretroviral therapy can effectively suppress HIV replication, but cannot achieve a cure because it is not capable of inactivating HIV genomes that are stably integrated into the host cell genome. We and others developed clustered regularly interspersed short palindromic repeat (CRISPR) strategies to target the HIV proviral genome, aiming to achieve permanent HIV inactivation. We previously reported complete virus control in a T cell line upon stable transduction with a lentiviral vector that encodes an antiviral CRISPR system. The designed antiviral guide RNAs (gRNAs) guide the endonuclease in a sequence-specific manner to the integrated HIV proviral genome and trigger an endonuclease attack. For the original Cas9 system, we needed to combine two gRNAs to achieve a cure.^1^^,^^2^ This was originally supposed to occur via excision of the HIV sequences between the two cleavage sites, but we documented that this route toward HIV inactivation is in fact only the minor route toward HIV inactivation. More frequently we did observe inactivated HIV genomes with indel mutations at both cleavage sites.^2^ We reasoned that this means that the target site that is cleaved first is closed by the cellular DNA repair machinery before the second target site is attacked. Upon a second DNA repair event, a process that is well-known to introduce mutations (indels), one ends up with an HIV genome with two mutated sites, which frequently causes virus inactivation as we intentionally target very conserved HIV sequences.

**Figure 1:**
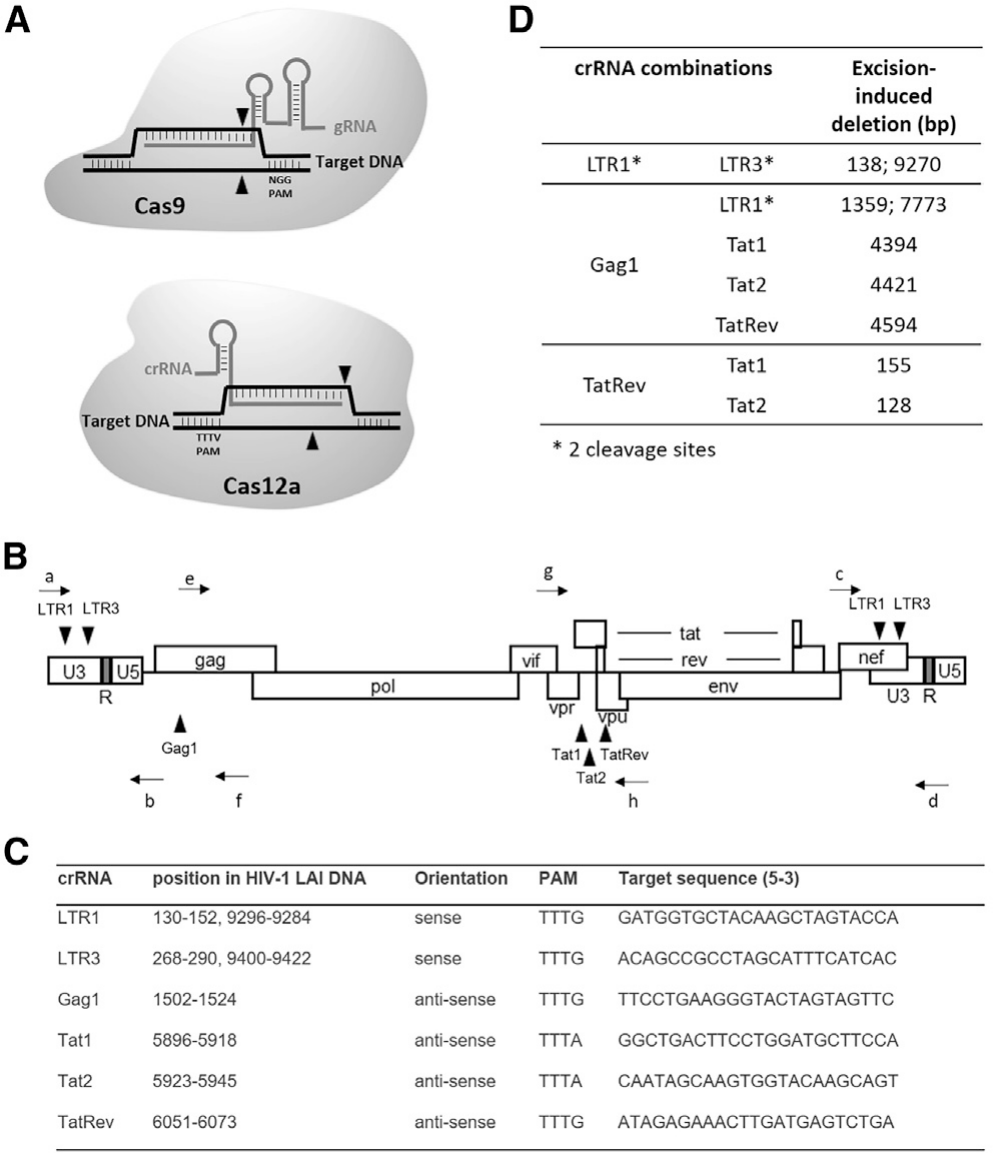
The crRNA targets for Cas12a attack on the HIV DNA genome (A) Mechanism of CRISPR-Cas9 and CRISPR-Cas12a-mediated genome engineering. The Cas9 nuclease with gRNA binds upstream of the PAM and induces a blunt-ended double-stranded break (DSB) exactly three base-pairs from the PAM. The Cas12a endonuclease with crRNA binds downstream of the PAM and induces a staggered-ended DSB distal from the PAM. The cleavage sites are marked as filled arrows. (B) Position of the antiviral gRNAs in sense (above) and antisense (below) orientation on the HIV proviral DNA. The position and direction of the PCR primers a−h is indicated by arrows. (C) The anti-HIV crRNAs and the actual targets sequences in the HIV genome. (D) The dual-crRNA combinations that were tested, with the distance between the two target sites indicated, which also reflects the size of the deleted fragment if excision occurs. Two copies of the LTR flank the HIV genome, thus doubling the number of target sites for LTR-targeting crRNAs (∗).

The CRISPR-Cas12a system with the associated CRISPR RNA (crRNA) was presented as a novel genome-editing tool that is more efficient and precise than the original CRISPR-Cas9 system.^3^, ^4^, ^5^ To recognize a double-stranded DNA (dsDNA) target, both the Cas9 and Cas12 endonucleases require a short sequence, termed the protospacer adjacent motif (PAM) next to the target site that facilitates gRNA and crRNA binding, respectively (Figure 1A). In contrast with the G-rich PAM of Cas9 targets, the Cas12a PAM is T-rich, thus significantly expanding the target range of the CRISPR family. With Cas9, target cleavage occurs immediately next to the PAM and usually leads to blunt-ended double-strand DNA breaks (DSB). In contrast, the Cas12a endonuclease creates DSBs with staggered ends and 5′ overhangs, and the cleavage site is located downstream at some distance from the PAM.^5^^,^^6^

Several studies have used the Cas12a nuclease for targeted genome editing in prokaryotes^7^ and eukaryotes,^8^, ^9^, ^10^, ^11^ but we are the first to test its ability to suppress HIV to reach a cure. In a previous study, we designed and tested multiple crRNA molecules against the HIV DNA genome.^12^ We measured modest HIV inhibition with Cas12a versus Cas9 in transient transfections, but Cas12a outperformed Cas9 in long-term HIV challenge studies in stably transduced T cells. We described a few antiviral crRNAs of the Cas12a system that were able to cure HIV-infected cultures when administered as a single antiviral, but virus escape was apparent for other crRNAs. To further improve this antiviral approach, we now set out to test combinations of two crRNAs for a combinatorial attack on HIV DNA, with the potential bonus of realizing permanent HIV inactivation through excision of the intervening HIV sequences as reported for Cas9.^2^^,^^13^^,^^14^ We now demonstrate that a dual crRNA attack does generally inactivate the HIV provirus genome more efficiently than the respective single crRNA attacks. Surprisingly, HIV DNA excision was hardly detected, revealing a differential outcome of a dual Cas9 versus Cas12a. These findings highlight the mechanistic differences between the two CRISPR systems, which will be discussed.

## Results

### Targeting the HIV genome with Cas12a and single versus dual crRNAs

The Benchling CRISPR Guide Design Software was previously used to design multiple crRNA molecules against the HIV DNA genome of the primary LAI virus isolate without apparent complementarity to cellular DNA sequences. We selected six crRNAs that target relatively conserved HIV sequences (Figures 1B and 1C). The crRNAs LTR1 and LTR3 target regulatory sequences in the long terminal repeat (LTR) promoter elements that flank the viral genome. These crRNAs target the U3 domain of the LTR promoter, but also the open reading frame that encodes the viral Nef protein. Of note, any LTR-targeting crRNA may trigger the excision of a large internal segment by cleaving at both LTRs, thus removing all protein-coding capacity.^15^ The other selected crRNAs (Gag1, Tat1, Tat2, and TatRev) target conserved HIV sequences that encode essential viral proteins. In fact, the TatRev crRNA targets two overlapping open reading frames. The antiviral crRNAs were tested individually and in pairwise combinations that could trigger excision of large provirus fragment (Figure 1D). Excision is considered an ideal HIV-inactivation and cure strategy. The crRNA combination LTR1+LTR3 targets HIV DNA at four positions, which may lead to the excision of the nearly complete proviral genome. A second crRNA set was based on Gag1, which was combined with either LTR1, Tat1, Tat2, or TatRev. In a third crRNA set, TatRev was combined with Tat1 or Tat2.

### HIV inhibition in transient transfections

**Figure 2:**
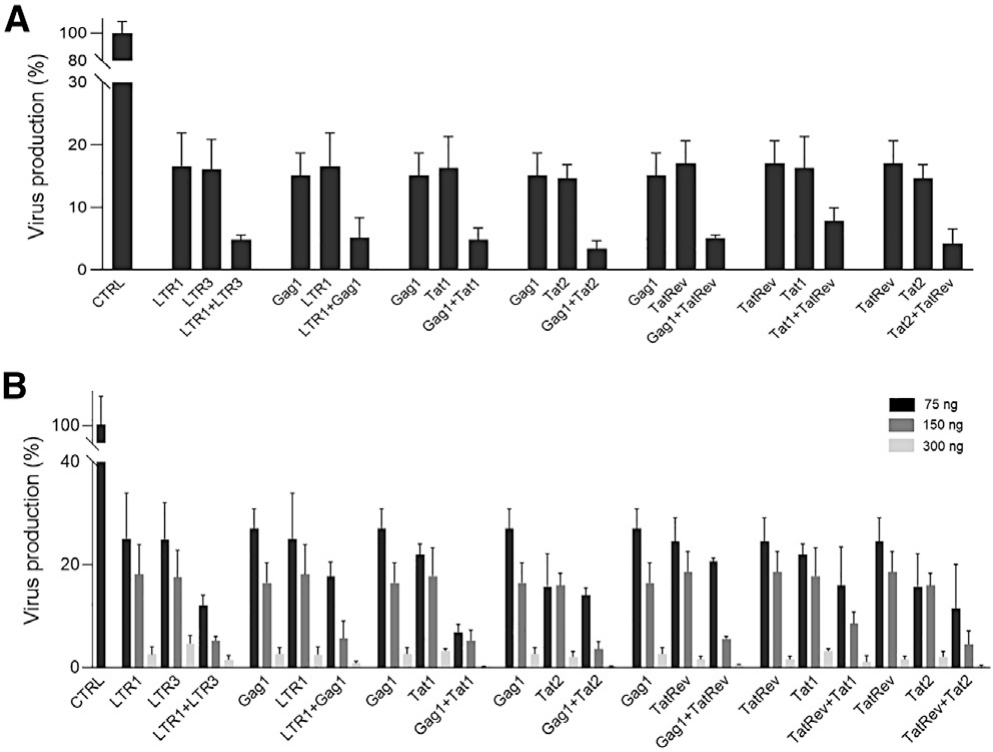
Antiviral activity of single and dual crRNAs tested in transfected HEK293T cells (A) The efficiency of the single and dual gRNAs to silence HIV DNA was tested in HEK293T cells transfected with plasmids expressing HIV LAI (300 ng), Cas12a and single crRNA (150 ng), or dual crRNAs (75 ng each). To quantify viral gene expression, the CA-p24 level was measured in the culture supernatant at two days after transfection. The CA-p24 level of the control transfection (CTRL) was set at 100%. (B) HEK293T cells were co-transfected with 300 ng of pLAI and an increasing amount of single or dual crRNA constructs (75, 150, and 300 ng). The CA-p24 level was determined at two days after transfection. Average values (± standard error of the mean) of three independent experiments are shown. Statistical analyses (two-way ANOVA followed by Tukey's post hoc test were performed, and differences among groups were considered significant when the corresponding p value was less than 0.05. All dual crRNA combinations resulted in a significant reduction of virus production compare with each crRNA individually.

We first tested these crRNA combinations versus the individual crRNA attacks in transient assays in which HIV LAI DNA (300 ng) is co-transfected in HEK293T cells with plasmids encoding the Cas12a endonuclease and a single or dual crRNAs. The single or dual crRNAs were used in equal amounts with 150 ng of total crRNA construct per transfection. We included a control crRNA (CTRL) that targets neither HIV nor the cellular genome. Virus production was measured by quantitation of the HIV CA-p24 protein in the culture supernatant at 2 days after transfection (Figure 2A). A high CA-p24 level was observed for CTRL, which was significantly decreased for the single crRNA antivirals. However, all dual crRNA combinations resulted in a further reduction, indicating improved HIV inhibition. We did not observe a correlation between the CA-p24 level and the actual number of target sites in HIV DNA (ranging from two to four), which could be due to the fact that profound inhibition was already obtained for the individual crRNAs. The inhibitory activity was subsequently tested in a titration with increasing amounts (75, 150, and 300 ng) of the crRNA constructs (Figure 2B). We observed a dose-dependent inhibition of HIV production for all crRNAs, either as single or dual mode.

### Efficient Cas12a-targeting of HIV-1 DNA with dual crRNAs

**Figure 3:**
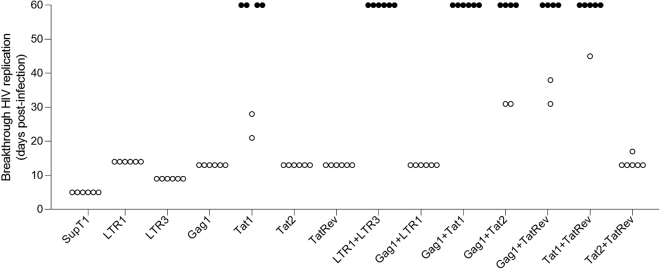
Monitoring HIV replication in single and dual crRNA transduced SupT1 cells Stably lentivirus-transduced SupT1 cells were infected with HIV (MOI of 0.3) and cultured for up to 60 days. The day at which massive virus replication (representing breakthrough HIV replication) was observed and each circle represents an independent culture (six per construct). Open circles represent the moment of virus break-through replication. Closed circles indicate candidate cured cultures that did not show any sign of virus replication up to day 60 when the experiment was stopped. SupT1 = non-transduced SupT1 cells. Please note that in our previous study we used 6.5-fold less HIV input to infect the same number of cells (Ref.^12^).

Next, we stably transduced the SupT1 T cell line with lentiviral vectors that encode all CRISPR components (Cas12a and a single or dual crRNAs). For each therapeutic cocktail, six independent cell cultures were infected with HIV LAI at a multiplicity of infection (MOI) of 0.3 and monitored the appearance of virus-induced syncytia, we scored unhindered HIV replication at day 5 for the six control cultures of non-transduced SupT1 cells yielded massive HIV-induced syncytia in all six cultures at day 5 (Figure 3). As reported before, HIV inhibition with an individual crRNA can be potent, but inhibition is usually only transient as virus escape occurs over time. This is exactly what we observed for most single crRNAs, with the exception of Tat1, which resulted in an apparent cure in four of the six cultures (closed circles in Figure 3). Most important, the combinatorial crRNA approach did profoundly improve the level of HIV inhibition, especially its durability. The weakest crRNA pairs are Gag1+LTR1 and Tat2+TatRev, where all six cultures demonstrated active HIV replication after 2 weeks. Such delayed replication compared with the SupT1 control is what we expect for viral escape. HIV can escape from Cas12a-mediated inhibition by mutation of the target sequence, which is caused by error-prone DNA repair upon Cas12a-mediated DNA cleavage.^12^ We did observe durable HIV suppression up to the end of the experiment at day 60 in all six cultures equipped with LTR1+LTR3 and Gag1+Tat1, which thus represent the strongest antiviral crRNA pairs. We did also observe durable HIV suppression in four of six Gag1+Tat2 and Gag1+TatRev cultures and five of six Tat1+TatRev cultures (closed circles in Figure 3). These apparently cured cultures represent the first examples of durable Cas12a-mediated HIV inactivation. We will first present the analysis of cultures that exhibited viral escape (open circles in Figure 3) and subsequently zoom in on the candidate-cured cultures (closed circles).

### Phenotype and genotype analysis of HIV escape candidates

**Figure 4:**
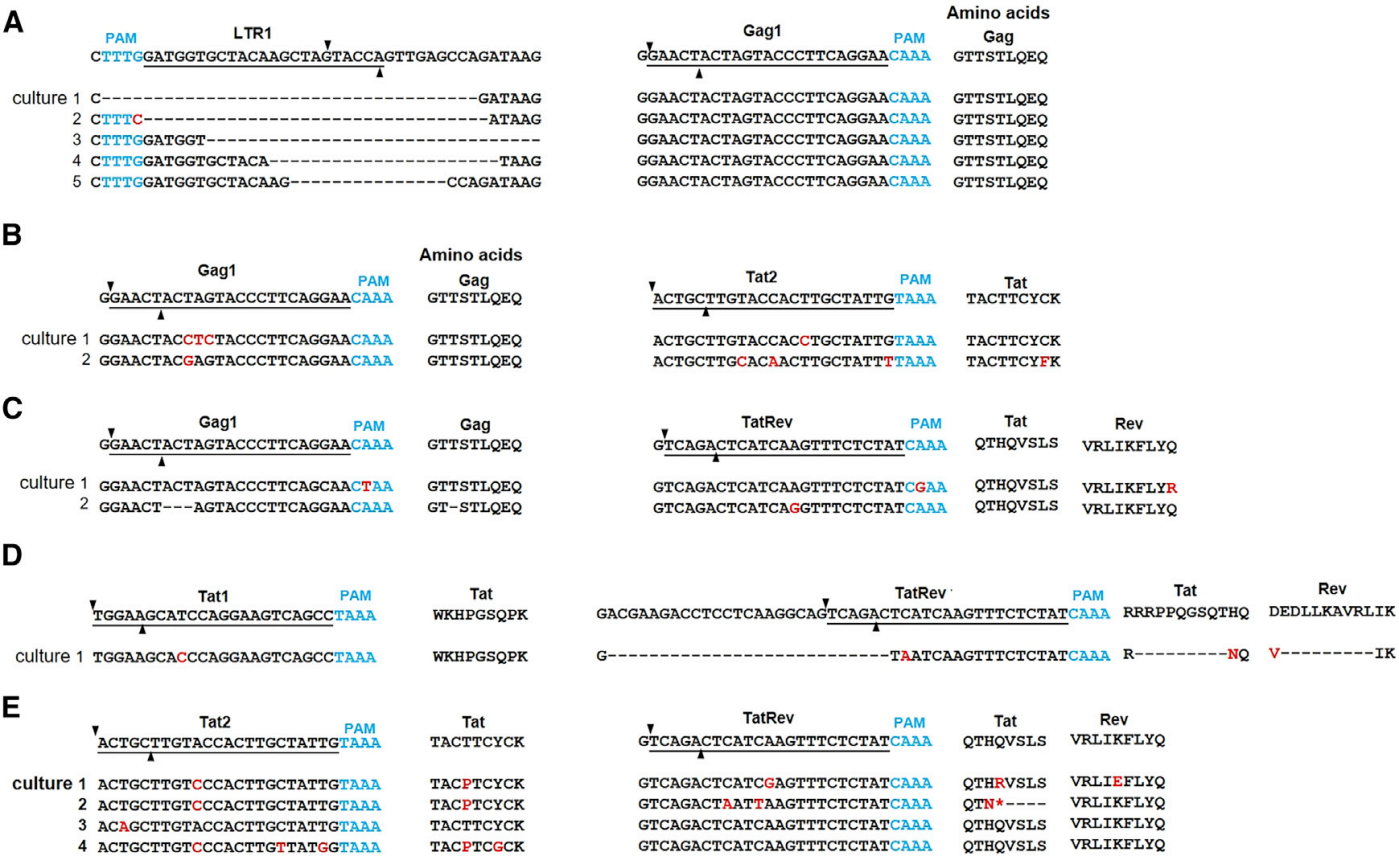
Analysis of crRNA-escape HIV variants (A) The LTR1+Gag1 crRNA combination instructs Cas12a to target the 5′/3′ LTRs and Gag region of HIV proviral DNA. (B–E) The crRNA combinations (Gag1+Tat2, Gag1+TatRev, Tat1+TatRev, and Tat2+TatRev) guide Cas12a to the respective genes in the HIV provirus. The supernatant of cells in which breakthrough virus replication was observed (Figure 3) was passaged onto fresh crRNA-protected cells, which were harvested at the peak of virus infection. The targeting regions were PCR amplified, Sanger sequenced, and subsequently aligned with the sequence of the input HIV LAI isolate (wild-type or WT shown at the top). The crRNA targets are underlined and the PAM is marked in blue. The DNA cleavage site is marked with black triangles, deletions are labeled with dashes, and substitutions are highlighted in red.

We performed a phenotypic test for the presence of crRNA-resistant HIV variants in these cultures by the passage of the cell-free virus onto a new batch of transduced cells (expressing the crRNA inhibitors). This step should purify the virus variants that acquired crRNA resistance. HIV replication was indeed observed in many cases, thus confirming the escape phenotype (Table S1). To demonstrate actual HIV escape, we isolated the cellular DNA as a source of integrated HIV proviruses and PCR-amplified and sequenced the HIV target regions. Figure 4 shows the sequence of the targets in different escape cultures as obtained by Sanger sequencing of the PCR product. Dual Gag1+LTR1 escape cultures displayed typical deletions in or across the Cas12a cleavage sites in the LTR1 target (underlined in Figure 4A). Note that such indels do not represent the typical HIV evolution pattern, but indels are standard for non-homologous end joining-mediated DNA repair. These target site mutations, sometimes even disrupting the PAM, hamper Cas12a recognition and consequently lead to HIV escape. As we reported before, insertions that are frequently seen upon Cas9-editing are absent in Cas12a lesions. We observed wild-type (WT) in the Gag1 site. This may indicate less efficient Cas12a-cleavage and evolutionary pressure at this site. As this phenotype test will specifically score replication-competent escape HIV variants, it is not surprising that we did not detect any gross Gag1-LTR1 excision events that are incompatible with HIV replication.

Point mutations were frequently selected in the targets in the Gag and Tat open reading frames (2× Gag1 and 2× Tat2 in the Gag1+Tat2 culture (Figure 4B), 1× Gag1 and 2× TatRev in Gag1+TatRev (Figure 4C), 1× Tat1 and 1× in TatRev (combined with a deletion) in Tat1+TatRev (Figure 4D), and 4× Tat2 and 2× TatRev in Tat2+TatRev (Figure 4E)). The absence of Cas12a-mediated deletions in these escape viruses is likely explained by the fact that such changes in the open reading frames are detrimental for virus replication. We sometimes observed multiple clustered point mutations and many of the observed mutations are silent in the respective open reading frame (e.g., three of the four Gag1 mutations, where the fourth has a triplet in-frame deletion, which underscores the evolutionary pressure to maintain the coding potential). Non-silent codon changes were more apparent for the Tat2 and TatRev targets. Silent mutations are much more difficult to realize in the TatRev gene overlap, thus forcing the virus to mutate essential protein functions. This could seriously reduce the viral replication capacity of these escape viruses. Strikingly, we observed an in-frame 27-nucleotide deletion in the TatRev target of a single Tat1+TatRev culture, which caused a deletion of nine amino acids in both reading frames (Figure 4D). This deletion did apparently allow viral escape, but viral replication was delayed up to day 23 (Table S1), which may suggest sub-optimal virus replication. Although this TatRev overlap is obviously constrained in its evolutionary possibilities, this domain (positions 6031–6056 in the HIV LAI genome) shows significant sequence variation across distinct HIV subtypes, consistent with the observed escape route.^16^

We next analyzed the acquired HIV sequences in more detail by TA-cloning. We chose two representative cultures of the crRNA combinations Gag1+LTR1 and Tat2+TatRev (Figures S1 and S2). For Gag1+LTR1, HIV sequences acquired deletions in or across the Cas12a cleavage site LTR1, while some “left-over” WT sequences were found for Gag1 as described above (Figure S1). We observed frequent disruption of the PAM motif in the LTR1 target (50% of sequences), which hampers Cas12a recognition and is likely to facilitate viral escape. Since Cas12a cleaves the target DNA outside the seed region (which is positioned a few nucleotides 3′ of the PAM where dsDNA-crRNA annealing is initiated), a likely explanation is that the target can undergo multiple rounds of cleavage and subsequent DNA repair, until a larger deletion is generated that destroys the PAM.^12^

For Tat2+TatRev escape viruses, most TA clones reveal Cas12a-introduced deletions in both Cas12a targets, which explains the escape phenotype (Figure S2). Again, we exclusively observed deletions or indels and no pure insertions, which represents the typical Cas12a signature. A certain percentage of WT sequence remained (10× Tat2 and 11× TatRev, marked by shadowing, of which 5× combined). We also observed small insertions that occurred in the context of a deletion, which we previously termed delins. We also detected a single excision event that removed all 147 nucleotides between the two Cas12a target sites (culture 1, bottom line in Figure S2). This obviously does not represent a replication-competent virus, but rather a defective proviral HIV genome.

Sometimes, the mutational pattern visible upon TA-cloning provides information on the order of events. For instance, HIV culture 1 with Tat2+TatRev first acquired the A-G substitution in the TatRev target (present in three clones marked as green m1 in Figure S2) and subsequently acquired different secondary hits in the Tat2 target. Likewise, HIV first acquired a four-nucleotide deletion in the Tat2 target (present in three clones marked as green −4) and subsequently added different hits in TatRev. Although the order of events can be verified, we assume that this order is random as dependent on a chance process at which of the two targets Cas12a-cleavage occurs first.

### Phenotype and genotype analysis of crRNA-cured HIV cultures

We next analyzed the candidate “cured” HIV cultures for the crRNA combinations LTR1+LTR3 (all six cultures), Gag1+Tat1 (all 6), Gag1+Tat2 (4 of 6), Gag1+TatRev (4 of 6), and Tat1+TatRev (5 of 6) that were able to block HIV replication up to the end of the culture period at day 60 (Figure 3). First, we performed an ultra-sensitive phenotype test for the presence of replication-competent virus by addition of unmodified SupT1 cells to samples of the treated cultures taken at day 30 and day 60 after infection as previously described.^1^ These cell mixtures are subsequently cultured for up to 30 days to monitor the presence of any trace of replication-competent virus. We scored the absence of any replicating virus for all day 60 samples and most day 30 samples, except for a single Gag1+TatRev and a single Tat1+TatRev culture (Table 1). These results demonstrate that most dual crRNA combinations prevent HIV escape and apparently cured these cell cultures. As such, dual therapy clearly outperforms a treatment with a single crRNA, except for some Tat1 cultures (Figure 3). This result does unequivocally demonstrate the superiority of the combinatorial cure approach.

**Table 1:** Replicating competent virus at 30 and 60 days after infection

crRNA	Culture	Day 30	Day 60
LTR1+LTR3	1	–	–
	2	–	–
	3	–	–
	4	–	–
Gag1+Tat1	1	–	–
	2	–	–
	3	–	–
	4	–	–
	5	–	–
	6	–	–
Gag1+Tat2	1	–	–
	2	–	–
	3	–	–
	4	–	–
Gag1+TatRev	1	–	–
	2	+	–
	3	–	–
Tat1+TatRev	1	+	–
	2	–	–
	3	–	–
	4	–	–

**Figure 5:**
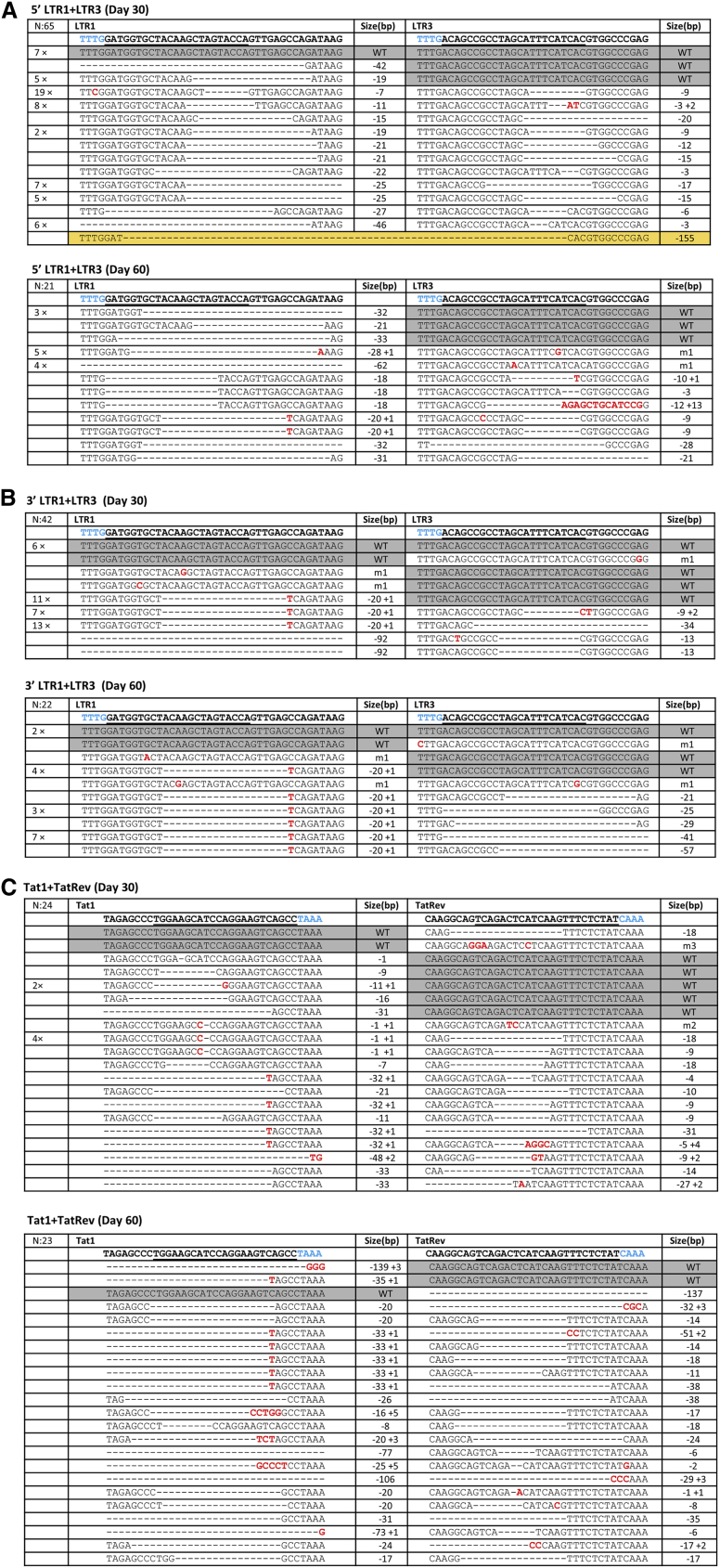
Proviral HIV DNA analysis in T cell cultures cured of replicating HIV (A–C) The LTR1+LTR3 crRNA combination targets the LTR repeats that were PCR-amplified with different primers and sequenced (5′ LTR in (A), 3′ LTR in (B and C)). The Tat1+TatRev crRNA combination targets the proviral Tat and Rev genes. The targeted regions were PCR-amplified, TA-cloned and sequenced at day 30 and day 60, respectively. The sequences were aligned with the input HIV LAI isolate. The crRNA targets are underlined and the PAM is marked in blue. Substitutions are shown in red. On the left we indicated the frequency of detection. On the right, we listed the size of the deletion (– sign), insertion (+ sign). Unedited WT sequences are marked with a gray background. A unique excision event that removes all HIV sequences between two cleavage sites in the 5′ and 3′ LTRs is highlighted with a yellow background.

We next performed an HIV genotype test for selected cell samples to map the lesions in the HIV genome that caused durable virus inactivation. We extracted the cellular DNA and PCR-amplified HIV genome regions, followed by TA-cloning and sequencing. Figures 5A and 5B show the 5′ and 3′ LTR targets in the LTR1+LTR3 cultures sampled at day 30 and 60, respectively. First of all, it is clear that both targets were frequently modified, such that most HIV genomes contain two LTR mutations. We observed the typical Cas12a-induced deletions and again no pure insertions were detected. Excision between the two Cas12a targets in the 5′ LTR was not frequently observed, except for a single event out of 65 TA-clones at day 30 (Figure 5A, upper panel, marked in orange). WT sequences were infrequently observed with 7 clones showing WT sequences in both the 5′ and 3′ LTR present at day 30. Importantly, we observed the complete loss of WT sequences in the LTR1 target (0 of 21 TA clones) at day 60, but some WT sequences remained in the LTR3 target. This means that no unaffected WT 5′ LTR sequence could be detected at the end of the culture period. Thus far, we only inspected the 5′ LTR for inactivating mutations, but it is likely that the proviral DNA acquired similar mutations in the 3′ LTR. Proviral 3′ LTR sequences of the same culture were analyzed with a different set of primers (Figure 5B). We observed a progressive loss of double WT-WT clones over time (six clones at day 30 and only two at day 60), but a few WT sequences remained in both LTR1 and LTR3 targets. Sometimes, the mutational pattern provides information on the order of the mutational events. For instance, the delin −20 + 1 in the LTR1 target seems to have been acquired first (31 sequences at day 30), followed by different LTR3 hits (Figure 5B).

Figure 5C shows the provirus analysis for Tat1+TatRev cultures at days 30 and 60. Again, most target sites are modified (deletions and delins), such that most HIV genomes contain two inactivating mutations. We observed a few leftover WT sequences in Tat1 (2×) and TatRev (6×) at day 30, but these loci were not genetically linked, which could have presented evidence for a fully WT virus. The number of unmodified WT sequences decreased progressively (1× in Tat1 and 2× in TatRev at day 60). A similar trend of accumulating mutations was observed for other crRNA combinations (e.g., Gag1+TatRev in Figure S3).

**Figure 6:**
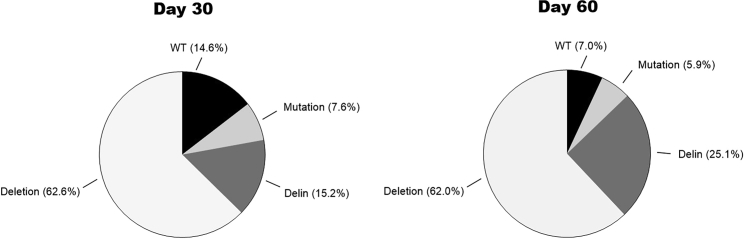
The mutational profile induced by Cas12a-mediated HIV-editing The frequency of different types of mutations (point mutations, deletions and delins) was calculated for all cultures that were cured of replicating HIV (4× Tat1, 6× LTR1+LTR3, 6× Gag1+Tat1, 4× Gag1+Tat2, 4× Gag1+TatRev, and 5× Tat1+TatRev). We show the day 30 and day 60 samples. Some unedited WT sequences were still present, especially in the early sample. Note that regular insertions are not generated by Cas12a-editing (Ref.^12^).

We finally calculated the relative frequency of the different types of mutations that we observed in all cultures that were apparently cured of replicating HIV (Figure 6). First, we observed the progressive loss of WT sequences (from 14.6% at day 30 to 7.0% at day 60), demonstrating ongoing HIV editing by Cas12a. We confirmed the absence of any regular insertions among the Cas12a-mutated sequences and there was a preference for regular deletions (62.6% early and 62.0% late) over delins (15.2 and 25.1%, respectively).

## Discussion

We set out to identify the best crRNA combinations for a double Cas12a attack on HIV DNA. Seven combinations were composed based on our previous description of potent crRNAs^12^ and two combinations turned out to perform optimally: LTR1+LTR3 and Gag1+Tat1. These combinations demonstrated potent and sustained antiviral activity in all six test cultures. We did not observe any virus escape and thus functionally cure the T cell culture of any replication-competent virus. We demonstrate that such a dual crRNA attack does ablate the HIV provirus genome more efficiently and durably than a single crRNA attack, where HIV escape occurred frequently. Complete HIV genomes upon single gRNA attack with Cas9 for 110 upon dual Cas9 attack with Cas12a for 60 days. Despite these superior *in vitro* HIV cure results for Cas12a compare to Cas9, several important issues could restrict its translation toward clinical use *in vivo*: e.g., how do we accurately deliver CRISPR to the right HIV reservoir cells, and how do we control its activity and limit off-target events.^17^^,^^18^ For instance, Murugan et al.^19^ recently indicated that Cas12a exhibits more off-target effects than previously appreciated.

Another important question concerns the inhibitory and cure efficacy of Cas12a against different HIV-1 strains and even subtypes. Two arguments do suggest that this will not be a major problem. First, minor mismatches are tolerated by the Cas12 system, thus ensuring cleavage of related HIV sequences. Second, we actually selected crRNAs against highly conserved viral target sequences to minimize inactivity against some virus variants. Still, some level of sequence variation will be present in exotic HIV strains and it is thus important to experimentally probe the activity spectrum against diverse viral strains and subtypes, like we did for Cas9.^19^

In this study, the combination of two crRNAs targeting the viral LTR domain (LTR1+LTR3) demonstrated potent antiviral activity and lead to the HIV cure in T cell cultures. These two crRNAs do actually target the HIV DNA at four positions because two LTRs flank the proviral genome. This LTR1+LTR3 combination could theoretically result in four DSBs, thus ideally fragmenting the HIV genome and triggering excision of most of the HIV provirus. One would also expect a relatively large excision of HIV sequences by the dual crRNA combinations Gag1+Tat1. However, a striking and unexpected result of this study is that such HIV-inactivating excisions were only sporadically observed in the Cas12a setting. HIV excision is the preferred outcome as no HIV protein-coding sequences remain. We previously tested a similar dual gRNA strategy in the Cas9 context and described more excision events, although excision remained a minor pathway toward HIV inactivation.^2^ Instead, the major route toward HIV inactivation by Cas9 is through a process that we termed hypermutation, in which one target is cleaved and repaired before the second cleavage event occurs, thus creating mutations at both target sites. Recent CRISPR studies in different fields described the same hypermutation phenomenon and DNA excision upon dual attack was absent or very rare.^20^, ^21^, ^22^ In terms of HIV inactivation, hypermutation is clearly less robust than HIV excision, but hypermutation is also likely to affect or even inactivate HIV replication as we target essential parts of the HIV genome.^1^ We also documented that the exact outcome (hypermutation versus excision) may differ for different gRNA pairs.^2^ We now provide strong evidence that the HIV-editing outcome also depends on the type of CRISPR system used. In contrast to the early Cas9 results, where excision represents 20%–40% of the introduced lesions,^2^ we hardly observed any excision events for Cas12a (just 2 cases out of 341 sequences analyzed). This obviously reduces the attractiveness of the Cas12a system for the generation of gross deletions in the HIV genome for permanent virus inactivation. Recent CRISPR studies in different fields observed the same hypermutation phenomenon and DNA excision was absent or very rare.^20^, ^21^, ^22^

We considered the possible molecular reason for this apparent difference in HIV excision capacity between the Cas9 and Cas12 endonucleases. Two mechanistic possibilities can be proposed. First and most likely, these two endonucleases will differ in the kinetics of DNA cleavage, which will affect the likelihood of a double-cleavage event that is required for excision. The outcome will basically depend on the kinetics of cleavage and subsequent DNA repair and the latter is known to be a very rapid process. We hypothesize that the cleavage kinetics may be more rapid for Cas9 than Cas12a, thus increasing the chance of introduction of a double cut, which is the required condition for excision of the intervening sequence. In this scenario, the more slowly acting Cas12a system will have the first cleaved target site repaired before the second target is cleaved, which will lead to hypermutation, that is the generation of indels at both targets.^3^

A second possibility is that the differential outcome is caused by the fact that these two endonucleases differ in the lesion that they leave upon DNA cleavage. Cas9 generates blunt-ended DSBs and Cas12a produces staggered-ended DSB. The nature of these DNA lesions will likely have a differential impact on the DNA repair kinetics and thus indirectly affect the chances of an excision event. Staggered DNA ends as generated by Cas12a are probably more likely to realign quickly in the process of DNA repair, thus preventing DNA excision.^23^^,^^24^ Biochemical and biophysical studies could shed further light on the underlying reasons for the absence of DNA excisions in double-crRNA editing by the Cas12a endonuclease. Anyhow, this editing system has clearly lost some of its attractiveness for use in an HIV cure cocktail because of the extremely low excision efficiency. But there may still be a future for the dual crRNA combinations that rapidly extinguished all infectious HIV in cell culture through hypermutation.

There may be other applications where the CRISPR-Cas12a system provides an attractive genome-editing platform with distinct properties. For instance, Cas12a may be helpful in research strategies where nucleotide insertions are not desired as this system does not introduce regular insertions. For instance, Cas12a may be helpful in research projects that try to avoid the creation of neo-epitopes in protein-encoding genes. Cas12a may also outperform Cas9 in strategies designed to disrupt the function of a gene as a somewhat larger deletion is induced by the use of a single crRNA.^12^ In addition, Cas12a has a higher sensitivity to mismatches in the gRNA than does Cas9; therefore, off-target sequence recognition will occur less frequently.^25^ Also, the relatively small size of the Cas12 gene could make delivery *in vivo* less challenging. Recently, genetic engineering has led to the development of smaller, but powerful Cas12 endonucleases like Cas12f (Cas14, 400–700 amino acids) and Cas12j (Cas Phi, 700–800 amino acids) which could facilitate efficient delivery *in vivo*.^25^, ^26^, ^27^, ^28^, ^29^ In general, we envisage that each application will require an optimally matching CRISPR system.

## Materials and methods

### Plasmid construction

The lentiviral plasmid pY109 (LentiCpf1, Addgene # 84,740) that harbors the LbCas12a gene and crRNA expression cassette was obtained from Feng Zhang.^30^ The lentiviral vector pLenti-SpBsmBI-sgRNA-Hygro (Addgene plasmid # 62205) that we used for the expression of gGag1 was a gift from Rene Maehr.^31^ The lentiviral plasmid pY109-HDV (crRNA1) was constructed as previously described.^12^ To construct plasmid pLenti-crRNA-Hygro-HDV (crRNA2), the crRNA scaffold with a 3′-terminal hepatitis delta virus (HDV) ribozyme was PCR-amplified from pY109-HDV and ligated into the plasmid pLenti-SpBsmBI-sgRNA-Hygro (Addgene plasmid # 62205) using BamHI and HpaI. The plasmid pLAI encodes the HIV primary CXCR4-using isolate LAI (subtype B).

### Cell culture, transfection, and transduction

Human embryonic kidney (HEK) 293T cells were cultured in DMEM (Life Technologies, Invitrogen, Carlsbad, CA) supplemented with 10% fetal calf serum (FCS), penicillin (100 U/mL) and streptomycin (100 mg/mL) in a humidified chamber at 37°C and 5% CO_2_. SupT1 T cells (ATCC CRL-1942) were grown in advanced RPMI (GIBCO BRL, Carlsbad, CA) supplemented with L-glutamine, 1% FCS, penicillin (30 U/mL), and streptomycin (30 mg/mL). For transient Cas12a/crRNA activity assays, HEK293T cells were transiently transfected using with Lipofectamine 2000 (Invitrogen) with 300 ng of pLAI and an increasing amount of the Cas12a/crRNA plasmids (75, 150, and 300 ng of pY109-HDV for single attack or 37.5, 75, and 150 ng of each crRNA plasmid (pY109-HDV and pLenti-crRNA-Hygro-HDV) for a combinatorial attack). Viral production in culture supernatant was quantified by an in-house developed CA-p24 ELISA. Lentivirus production in HEK293T cells was performed as previously described.^32^ Briefly, HEK293T cells (at approximately 80% confluence in a six-well plate) were transfected with the lentiviral plasmid pY109-HDV or pLenti-crRNA-Hygro-HDV and packaging plasmids pSYNGP, pRSV-rev and pVSV-g using Lipofectamine 2000. Two days after transfection, the lentiviral vector containing supernatant from two wells was centrifuged at a low speed, filtered (0.45 μm), and concentrated to 150 μL using ultra-4 centrifugal filter units (Merck Millipore Amicon, Kenilworth, NJ). SupT1 cells (4×10^5^ cells in 1 mL of culture medium) were transduced with the concentrated lentiviral vectors. After transduction, the cells were cultured in the presence of puromycin (1 μg/mL) for 10 days to select SupT1 cells expressing Cas12a, crRNA1, and hygromycin (500 μg/mL) for 14 days to select cells expressing crRNA2.

### HIV production and infection

To produce HIV LAI, HEK293T cells were transfected with 5 μg pLAI using Lipofectamine 2000 (Invitrogen). Two days after transfection, the culture supernatant was collected, filtered (0.45 μm) and aliquoted. The MOI was determined. An equal amount of non-transduced SupT1 cells and transduced SupT1 cells (2×10^5^ cells in 1 mL medium) were infected with HIV (MOI 0.3) in six parallel cultures for each group. The infected cells were cultured for 60 days and passaged twice a week. Virus replication was evaluated by scoring syncytia formation when cells were passaged.

### HIV phenotype and genotype assay

To analyze the cultures that showed “breakthrough virus replication” for candidate escape viruses, the culture supernatant was passaged onto fresh crRNA-transduced SupT1 cells to confirm the escape phenotype (phenotype assay). Total cellular DNA (with integrated HIV proviruses) was isolated at the peak of the infection with the QIAGEN DNAeasy kit and worked up for sequencing (genotype assay). For cured cultures that did not demonstrate breakthrough virus replication at 30 and 60 days after infection, we mixed the culture sample with an equal number of control (non-transduced) SupT1 cells, followed by culturing to monitor the formation of virus-induced syncytia (phenotype assay). When the absence of any replicating competent virus was confirmed, total cellular DNA was isolated with the QIAGEN DNAeasy kit and worked up for sequencing (genotype assay). The crRNA target regions were amplified by PCR with the primers listed in Table S2. Figure 1B shows the position of the primers in the HIV genome. Primers a + b and c + d are used to amplify 5′ and 3′ LTR fragments, respectively. Primers e + f and g + h detect Gag and Tat target sites, respectively. Primers a + f amplify the LTR + Gag region. The PCR products were gel-purified, cloned in the TA-cloning vector and multiple TA-cloned fragments were analyzed by Sanger sequencing. Sequences were aligned with the WT HIV pLAI sequence.

## Supplemental information

Document S1. Figures S1–S3 and Tables S1 and S2

Document S2. Article plus supplemental information
