# Supplementary material for: A combinatorial CRISPR-Cas12a attack on HIV DNA
Source: Mol Ther Methods Clin Dev. 2022 Feb 26;25:43–51. doi: 10.1016/j.omtm.2022.02.010 (PMC8933334; doi:10.1016/j.omtm.2022.02.010)
Supplement: Document S1. Figures S1–S3 and Tables S1 and S2 [file mmc1.pdf]

OMTM, Volume *25*

Supplemental information

A combinatorial CRISPR-Cas12a attack on HIV DNA

Minghui Fan, Ben Berkhout, and Elena Herrera-Carrillo

Gag1+LTR1 Culture 1

N:23	LTR1		Gag1	
	TTTGGATGGTGCTACAAGCTAGTACCAGTTGAGCCAGATAAG		AGCAGGAACTACTAGTACCCTTCAGGAACAAA	
	TTTGGATGGTGCTACGAGC---------TTGAGCCAGATAAG	-10 +5	AGCAGGAACTACTAGTACCCTTCAGGAACAAA	WT
10 ×	---------------------------------CCAGATAAG	-42	AGCAGGAACTACTAGTACCCTTCAGGAACAAA	WT
	---------------------------------CCAGATAAG	-42	AGCAGGAACTACTAGTACCCTTCAGGAACTAA	m1
	---------------------------------CCAGATAAG	-42	AGCAGGAACTACTAGCACCCTTCAGGAACAAA	m1
2 ×	TTTGGATGGTGCTATGAGC---------TTGAGCCAGATAAG	-10 +5	AGCAGGAACTAGTA---CCCTTCAGGAACAAA	-3
3 ×	---------------------------------CCAGATAAG	-42	AGCAGGAACTAGTA---CCCTTCAGGAACAAA	-3
5 ×	---------------------------------CCAGATAAG	-42	AGCAGGAA--------------------CAAA	-20

Gag1+LTR1 Culture 2

N:23	LTR1		Gag1	
	TTTGGATGGTGCTACAAGCTAGTACCAGTTGAGCCAGATAAG		AGCAGGAACTACTAGTACCCTTCAGGAACAAA	
	TTTGGATG------------------------------AAAG	-30 +1	AGCAGGAACTACTAGTACCCTTCAGGAACAAA	WT
	TTTGGATGGTGC-----------------------------G	-28	AGCAGGAACTACTAGTACCCTTCAGGAACAAA	WT
	TTTCTTTG-------------------GTTGAGCCAGATAAG	-19 +5	AGCAGGAACTACTAGTACCCTTCAGGAACAAA	WT
5 ×	TTTGGATGGTGCTACAAG---------------CCAGATAAG	-15	AGCAGGAACTACTAGTACCCTTCAGGAACAAA	WT
3 ×	TTTGGATGGTGCTACAAG------------------------	-24	AGCAGGAACTACTAGTACCCTTCAGGAACAAA	WT
	TTTGGATGGTGCTACAAG---------------CCAGATAAG	-15	AGCAGGAGCTACTAGTACCCTTCAGGAACAAA	m1
	TTTGGATGGTGCTACAAG---------------CCAGATAAG	-15	AGCGGGAACTACTAGTACCCTTCAGGAACAAA	m1
	TTTGGATGGTGCTACAAG------------------------	-24	AGCAGGAACTACTAGTGCCCTTCAGGAACAAA	m1
	TTTGGATGGTGCTACAAG---------------CCAGATAAG	-15	AGCAGGAACTACTAGTACCCT-CAGGAACAAA	-1
	TTTCTTTG-------------------GTTGAGCCAGATAAG	-19 +5	AGCAGGAACTA---GTACCCTTCGGGAACAAA	-3
	TTTGGATGGTGCTACAAG---------------CCAGATAAG	-15	AGCA------------ACCCTTCGGGAACAAA	-12
	TTTGGATGGTGCTACAAG---------------CCAGATAAG	-15	AGCAGGAACT----------TTCAGGAACAAA	-10
	TTTGGATGGTGCTACAAG---------------CCAGATAAG	-15	AGC-----------GTACCCTTCAGGAACAAA	-11
	TTTGAATGGTGCTACAAG---------------CCAGATAAG	-15	AGCAGG---TACTAGTACCCTTCAGGAACAAA	-3
	TTTGAATGGAGCGACA----------------GCCAGATAAG	-16	AGCAGGAACT----------TTCAGGAACAAA	-10
	TTTGGATG------------------------------AAAG	-30 +1	AGCAG-AACTACTAGTACCCTTCAGGAACAAA	-1
	TTTGGATGGTGCTACAAG---------------CCAGATAAG	-15	AGCAA--------AGTACCCTTCAGGAACAAA	-8 +1

Figure S1. **Sequence analysis of HIV proviral DNA from two representative cultures in Gag1+LTR1 crRNA-protected SupT1 cells, related to Figure 4.** The crRNA-targeted region was amplified by PCR and TA-cloned. Multiple TA clones were sequenced. Sequences were aligned to the wild-type HIV LAI isolate, which is the wild-type (WT) shown at the top. The crRNA targets are underlined and the PAM is marked in blue. Deletions were labelled with dashes. Substitutions are highlighted in red. In front of the sequences, we indicated those that were detected multiple times. Unedited WT sequences were marked in grey background.

Tat2 + TatRev culture 1

N: 39	Tat2	Size (bp)	TatRev	Size (bp)
	TAAAACTGCTTGTACCACTTGCTATTGTAAA		AAGGCAGTCAGACTCATCAAGTTTCTCTATCAAA	
5 ×	TAAAACTGCTTGTACCACTTGCTATTGTAAA	WT	AAGGCAGTCAGACTCATCAAGTTTCTCTATCAAA	WT
	TAAAACTGCTTGTACCACTTGCTATTGTAAA	WT	AAGGCAGTCAGACTCATCGAGTTTCTCTATCAAA	m1
	TAAAACTGCTTGTACCACTTGCTATTGTAAA	WT	AAGGCAGTCA–CTCATCAACTTTCTCTATCAAA	-2
2 ×	TAAAACTGCTTGTACCACTTGCTATTGTAAA	WT	AAGGCAGTCA------TCAAGTTTCTCTATCAAA	-6
	TAAAACTGCTTGTACCACTTGCTATTGTAAA	WT	AAGGCAGTC------------------TATCAAA	-18
4 ×	TAAAACTGCTTGTCCCACTTGCTATTGTAAA	m1	AAGGCAGTCAGACTCATCAAGTTTCTCTATCAAA	WT
	TAAAACTGCT-CTACCACTTGCTATTGTAAA	-1 +1	AAGGCAGTCAGACTCATCAAGTTTCTCTATCAAA	WT
	TAAAACTGC----ACCACTTGCTATTGTAAA	-4	AAGGCAGTCAGACTCATCAAGTTTCTCTATCAAA	WT
	TAAAACTGC-CATACCACTTGCTATTGTAAA	-1 +2	AAGGCAGTCA------TCAAGTTTCTCTATCAAA	-6
2 ×	TAAAACTGCTT-TACCACTTGCTATTGTAAA	-1	AAGGCAGTCA------TCAAGTTTCTCTATCAAA	-6
	TAAAACTGCT-GTACCACTTGCTATTGTAAA	-1	AAGGCAGTCA------TCAAGTTTCTCTATCAAA	-6
	TAAAAC---------GACTTGCTATTGTAAA	-9 +1	AAGGCAGTCA------TCAAGTTTCTCTATCAAA	-6
	TAAAA---GCTGTACCACTTGCTATTGTAAA	-3 +2	AAGGCAGTCAGACTCATCGAGTTTCTCTATCAAA	m1
	TAAAACTGCTT-TACCACTTGCTATTGTAAA	-1	----------------TCAAGTTTCTCTATCAAA	-18
	TAAAACTGC----ACCACTTGCTATTGTAAA	-4	AAGGCAGTCA------TCAAGTTTCTCTATCAAA	-6
	TAAAACTGCTT--ACCACTTGCTATTGTAAA	-2	AAGGCAGTCA------TCAAGTTTCTCTATCAAA	-6
	TAAAACTGCTTG---------CTATTGTAAA	-9	AAGGCAGTCAGA--------GTTTCTCTATCAAA	-9
	TAAAACTGC----ACCACTTGCTATTGTAAA	-4	AAGGCAGTC---------AAGTTTCTCTATCAAA	-9
	TAAA---------CCCACTTGCTATTGTAAA	-9 +1	AAGGCAGTCA------TCAAGTTTCTCTATCAAA	-6
	TAAAA------GGCAAACTTGCTATTGTAAA	-6 +5	AAGGCAGTC---CTCATCAAGTTTCTCTATCAAA	-3
	-----------------CTTGCTATTGTAAA	-25	AAGGCAGTC--------CAAGTTTCTCTATCAAA	-8
	TAAAACTG----TACCACTTGCTATTGTAAA	-4	AAGGCAGTCAGACTCATCGAGTTTCTCTATCAAA	m1
	TAAAACTG----TACCACTTGCTATTGTAAA	-4	AAGGCAGTCAGA--------GTTTCTCTATCAAA	-9
	TAA---------------GGGCTATTGTAAA	-15 +2	AAGGCAG-----GACATCAAGTTTCTCTATCAAA	-5
	TA---------------CTTGCTATTGTAAA	-15	AAGGCAGTCA---------AGTTTCTCTATCAAA	-9
	TAAAACTG----TACCACTTGCTATTGTAAA	-4	AAGGCAGTC----TCATCAAGTTTCTCTATCAAA	-4
	TAAAA-------TACCACTTGCTATTGTAAA	-7	AAGGCAGTCA------TCAAGTTTCTCTATCAAA	-6
	TAAA---------CCCACTTGCTATTGTAAA	-9 +1	---------------------TTTCTCTATCAAA	-18
	T----------GTACCACTTGCTATTGTAAA	-10	AAGGCAGTCA------TCAAGTTTCTCTATCAAA	-6
	TAAAA---TATCAAA			-147

Tat2 + TatRev culture 2

N:35	Tat2	Size (bp)	TatRev	Size (bp)
	CCTAAAACTGCTTGTACCACTTGCTATTGTAAA		AAGGCAGTCAGACTCATCAAGTTTCTCTATCAAA	
15 ×	CCTAAAACTGCTTGTACCACTTGCTATTGTAAA	WT	AAGGCAGTCAGACTCATCAAGTTTCTCTATCAAA	WT
	CCTAAAACTGCTTGTACCACTTGCTATTGTAAA	WT	AAGGCAGTCA------TCAAGTTTCTCTATCAAA	-6
	CCTAAAACTGCTTGTACCACTTGCTATTGTAAA	WT	AAGGCAGTCAGACTCATC---TTTCTCTATCAAA	-3
	CCTAAAACTGCTTGTACCACTTGCTATTGTAAA	WT	-------------------AGTTTCTCTATCAAA	-25
2 ×	CCTAAAA----TTGTACCACTTGCTATTGTAAA	-4	AAGGCAGTCAGACTCATCAAGTTTCTCTATCAAA	WT
	CCTA---------GTACCACTTGCTATTGTAAA	-9	AAGGCAGTCAGACTCATCAAGTTTCTCTATCAAA	WT
	CCTA---------------CTTGCTATTGTAAA	-15	AAGGCAGTCAGACTCATCAAGTTTCTCTATCAAA	WT
	----------------CCACTTGCTATTGTAAA	-18	AAGGCAGTCAGACTCATCAAGTTTCTCTATCAAA	WT
3 ×	CCTAAAACTGC----ACCACTTGCTATTGTAAA	-4	AAGGCAGTCAGACTCA---AGTTTCTCTATCAAA	-3
	----------------CCACTTGCTATTGTAAA	-18	AAGGCAGTCAGACTCATC---TTTCTCTATCAAA	-3
	CCTAAAA-----GTTACCACTTGCTATTGTAAA	-5 +2	-----------------------------TCAAA	-28
	CCTAAAACT------------TGCTATTGTAAA	-12	AAG------------------TTTCTCTATCAAA	-18
	CCTAAA--------TACCACTTGCTATTGTAAA	-8	AAGGCAGTCAGACTCATCGAGTTTCTCTATCAAA	m1
	CCTAAAA--------------AGTTATTGTAAA	-14 +3	AAGGCAGTCAGACTC---GAGTTTCTCTATCAAA	-3 +1
	CCTAAAA------------CTTGCTATTGTAAA	-12	A----------------------------TCAAA	-28
	CCTAAAACTGCTT-TACCACTTGCTATTGTAAA	-1	AAGGCAGT---------------------TCAAA	-21
	CCT--------TTGTACCACTTGCTATTGTAAA	-8	AAGGCAGT--------------TTCTCTATCAAA	-14
	-----------AGTTACCACTTGCTATTGTAAA	-16 +3	AAGGCA------------AAGTTTCTCTATCAAA	-12

Figure S2. **Sequence analysis of HIV proviral DNA from two representative cultures in Tat2-TatRev crRNA-protected SupT1 cells, related to Figure 4.** The crRNA-targeted region was amplified by PCR and TA-cloned. Multiple TA clones were sequenced. Sequences were aligned to the wild-type HIV LAI isolate, which is the wild-type (WT) shown at the top. The crRNA targets are underlined and the PAM is marked in blue. Deletions were labelled with dashes. Substitutions are highlighted in red. Unedited WT sequences were marked in grey background. Excision sequences between two cleavage sites were marked in yellow background. In front of the sequences, we indicated those that were detected multiple times.

N: 27	Gag1	Size(bp)
	AGCAGGAACTACTAGTACCCTTCAGGAACAAA	
4 ×	AGCAGGAACTACTAGTACCCTTCAGGAACAAA	WT
	AGCAGGAACTACTAGTACCCTTCAGGGACAAA	m1
8 ×	AGCAGGAACTA---GTACCCTTCAGGAACAAA	-3
	AGCAGGAAC---AAGTACCCTTCAGGAACAAA	-3 +1
	CGCAGGAACTA---GTACCCTTCAGGAACAAA	-3
	AGCAGGAACT----GTACCCTTCAGGAACAAA	-4
	AGCA------CGGAGTACCCTTCAGGAACAAA	-6 +3
	AGCAGGA-------GTACCCTTCAGGAACAAA	-7
	AGCAGG--------TTACCCTTCAGGAACAAA	-8 +1
2 ×	AGCAGG--------GTACCCTTCAGGAACAAG	-8
	AG---------TTAGTACCCTTCAGGAACAAA	-9 +1
	AGCAG----------TACCCTTCAGGAACAAA	-10
	AG-------------TACCCTTCAGGAACAAA	-13
	AGCAGGAAC--------------------AAA	-20
	--------------------------------	-47
	--------------------------------	-60

N: 18	Gag1	Size(bp)
	AGCAGGAACTACTAGTACCCTTCAGGAACAAA	
	AGCAGGAACTACTAGCACCCTTCAGGAACAAA	m1
	AGCAGGAACTA--AGTACCCTTCAGGAGCAAA	-2
3 ×	AGCAGGAACTA---GTACCCTTCAGGAACAAA	-3
	AGCAG-----TCGCGTACCCTTCAGGAACAAA	-5 +4
	AGCAGGAACT----GTACCCTTCAGGAACAAA	-4
	AGCAGGA-------GTACCCTTCAGGAACAAG	-7
	AGC-------CCTAGTACCCTTCAGGAACAAA	-7 +1
	AGCAGTA----------CCCTTCAGGAACAAA	-10
	AG-------------TACCCTTCAGGAACAAA	-13
	AGCA-----------TACCCTTCAGGAACAAA	-11
	AGCAGGGA-------------------ACAAA	-19
	AGCAGG--------------------AACAAA	-20
	------------------------------GT	-41 +2
	--------------------------------	-44
	--------------------GTCAGGAACAAA	-23 +1
	-----------------------GAGAACAAA	-26 +2

N: 19	Tat1	Size(bp)
	GAGCCCTGGAAGCATCCAGGAAGTCAGCCTAAA	
5 ×	GAGCCCTGGAAGCATCCAGGAAGTCAGCCTAAA	WT
	GAGCCCTGGAAGCATCCAGGAAGTCAGCCTAAG	m1
	GAGCCCTGGAAGCATCCAGGTAGTCAGCCTAAA	m1
	GAGCCCTGGAAGCACCCAGGAAGTCAGCCTAAG	m2
3 ×	GAGCCCTGGAAG-CCCAGGAAGTCAGCCTAAA	-2 +1
	GAG----------------GAAGTCAGCCTAAA	-16
	GAGCCCT--------CCAGGAAGTCAGCCTAAA	-8
	GAGCCCT----------AGGAAGTCAGCCTAAA	-10
2 ×	-------------------GAAGTCAGCCTAAA	-31
2 ×	------------------------TAGCCTAAA	-32 +1
	-----------------------------AGTG	-50 +4

N: 19	Tat1	Size(bp)
	TAGAGCCCTGGAAGCATCCAGGAAGTCAGCCTAAA	
	TAGAGCCCTGGAAGCACCCAGGAAGTCAGCCTAAA	m1
	TAGAGCCCTGGAAGCCTACAGGAAGTCAGCCTAAA	m2
	TAGAGCCCTGGGAGCATCCAGGAAGTCAGCCTAAA	m1
2 ×	TAGAGCCCT--------CCAGGAAGTCAGCCTAAA	-8
5 ×	TAGAGCCCT----------AGGAAGTCAGCCTAAA	-10
	TATAGCCCT----------AGGAAGTCAGCCTAAA	-10
	TAGAG----------TGCTAGGAAGTCAGGCTAAA	-10 +4
3 ×	--------------------------TAGCCTAAA	-32 +1
	--------------------------TGGCCTAAA	-32 +2
	---------------------GAAGTCAGCCTAAA	-31
	-----------------------------------	-52
	TAGAGCCCT-----------------CAGCCTAAA	-17

Gag1+Tat2 day 30

N: 23	Gag1	Size (bp)
	AGCAGGAACTACTAGTACCCTTCAGGAACAAA	
6 ×	AGCAGGAACTACTAGTACCCTTCAGGAACAAA	WT
	AGCAGGAACTA--GGTACCCTTCAGGAACAAA	-2 +1
	AGCGGGAACTA--AGTACCCCTCAGGAACAAA	-2
	AGCA---TGAACTAGTACCCCTCAGGAACAAA	-3 +3
3 ×	AGCAGGAACTA---GTACCCTTCAGGAACAAA	-3
	AGCAGG--------TTACCCTTCAGGAACAAA	-8 +1
	AGCA-------------CCCTTCAGGAACAAA	-13
4 ×	AG--------------------CAGGAACAAA	-20
	AG-------------TACCCTTCAGGAACAAA	-13
	AGC-------------------CAGGAACAAG	-19
	AGCAGGAACTACTACAA---------------	-16 +3
	---------------------------ACAAA	-30
	---------------------------ACAAA	-52

N: 23	Tat2	Size (bp)
	TAAAACTGCTTGTACCACTTGCTATTGTAAA	
7 ×	TAAAACTGCTTGTACCACTTGCTATTGTAAA	WT
	TAAAACTGTTTGTACCACTTGCTATTGTAAA	m1
2 ×	TAAAACTGCTTGTACCAATTGCTATTGTAAA	m1
2 ×	TAAAACTGCTTGTACCAAATGCTATTGTAAA	m2
	TAAAACTGC--GTACCACTTGCTATTGTAAA	-2
2 ×	TAAAAC----TGTACCACTTGCTATTGTAAA	-4
3 ×	TAAA-----TTGTACCACTTGCTATTGTAAA	-5
	-----------------CTTGCTATTGTAAA	-20
	---------------------------CGGG	-32 +4
	-------------------------------	-36
	--------------ATACTTGCTATTGTAAA	-36 +2
	-----------------------ATTGTAAA	-39

Gag1+Tat2 day 60

N: 20	Gag1	Size (bp)
	AGCAGGAACTACTAGTACCCTTCAGGAACAAA	
	AGCAGGAACTACTAGTACCCTTCAGGAACAAA	WT
	AGCAGGAACTA--AGTACCCTTCAGGAACAAA	-2
4 ×	AGCAGGAACTA---GTACCCTTCAGGAACAAA	-3
	AGCAGGAACTA---ATACCCTTCAGGAACAAC	-3 +1
	AGCA------CGGAGTACCCTTCAGGAACAAA	-6 +3
	AG--------TCTAGTACCCTTCAGGAACAAA	-8 +1
	AGCAG----------TACCCTTCAGGAACAAA	-10
2 ×	AG-------------TACCCTTCAGGAACAAA	-13
	-----------CTAGTACCCTTCAGGAACAAA	-11
	AG-------------GAACAAATAGGAACAAA	-13 +8
3 ×	AGCAGGAA--------------------CAAA	-20
	AG----------------------GGAACAAA	-22
	--------------------------------	-36
	---------------AGGCCCTTCAGGAACAAA	-104 +3

N: 27	Tat2	Size (bp)
	TAAAACTGCTTGTACCACTTGCTATTGTAAA	
	TAAGACTGCTTGAACCAATTGCTATTGTAAA	m3
4 ×	TAAAACTGCTT-TACCACTTGCTATTGTAAA	-1
	TAAAACTGC--GTACCACTTGCTATTGTAAA	-2
2 ×	TAAAACTGCTT--ACCACTTGCTATTGTAAA	-2
	TAAAACTAAT---ACCACGTGCTATTGTAAA	-3 +3
2 ×	TAAAACTG----TACCACTTGCTATTGTAAA	-4
6 ×	TAAA-----TTGTACCACTTGCTATTGTAAA	-5
	TAA-----CTTGTACCACTTGCTACTGTAAA	-5
	TAAA--------TGCCACTTGCTATTGTAAA	-8 +2
	TAAAAC---------CACTTGCTATTGTAAA	-9
	TA---------GTACCACTTGCTATTGTAAG	-9
	-------------TACCACTTGCTATTGTAAA	-13
2 ×	--------ACCTTACCACTTGCTATTGTAAA	-14 +4
	TAA--------------CTTGCTATTGTAAA	-14
	TAAA-------------------------AA	-24
	TA---------------------------GA	-27 +1

Gag1+TatRev day 30

N: 20	Gag1	Size (bp)
	AGCAGGAACTACTAGTACCCTTCAGGAACAAA	
4 ×	AGCAGGAACTACTAGTACCCTTCAGGAACAAA	WT
5 ×	AGCAGGA---ACTAGTACCCTTCAGGAACAAA	-3
	AGCAGG---GACTAGTACCCTTCAGGAACAAA	-3
	AGCAGG--------GTACCCTTCAGGAACAAA	-8
	AG----------------TTTTCAGGAACAAA	-16 +2
	A----------CTAGTACCCTTCAGGAACAAA	-10 +2
	AG-----------AGTACCCTTCGGGAACAAA	-11
3 ×	AG-----------AGTACCCTTCAGGAACAAA	-11
	AG--------------------CAGGAACAAA	-20
	--------------------------------	-35
	---------------------------ACAAA	-52

N: 20	TatRev	Size (bp)
	CAAGGCAGTCAGACTCATCAAGTTTCTCTATCAAA	
7 ×	CAAGGCAGTCAGACTCATCAAGTTTCTCTATCAAA	WT
2 ×	CAAGGCAGTCAGAC--ATCAAGTTTCTCTATCAAA	-2
	CAAGGCAGTC----TCATCAAGTTTCTCTATCAAA	-4
2 ×	CAAGGCAGTCA---------AGTTTCTCTATCAAA	-9
	CAAGGCA-------TCATCAAGTTTCTCTATCAAA	-7
	CAAGGCAGTCAGA----TTAAGTTTCTCTATCAAA	-4 +2
	-------------------TCAAGTTTCTCTATCAAA	-19
	CA------------------------TCTATCAAA	-24
	CAA---------------------------TCAAA	-27
	CAAG--------------------------TCAAA	-26
	---------------------GTTTCTCTATCAAA	-30
	--------------------------AGGATCAAA	-32 +3

Gag1+TatRev day 60

N: 19	Gag1	Size (bp)
	AGCAGGAACTACTAGTACCCTTCAGGAACAAA	
4 ×	AGCAGGAACTA---GTACCCTTCAGGAACAAA	-3
	AGCAGGAGCTA---GTACCCTTCAGGAACAAA	-3
	AGCCGGAACTA---GTACCCTTCAGGAACAAA	-3
	AGCAGG-----CTAGTACCCTTCAGGAACAAA	-5
	A----------CTAGTACCCTTCAGGAACAAA	-10
2 ×	AGC----------AGTACCCTTCAGGAACAAA	-10
	AGCAGGAACT-------CCCTTCAGGAACAAA	-7
	TGCA-----------TACCCTTCAGGAACAAA	-11
	AGCAGG--------TTACCCTTCAGGAACAAA	-8 +1
	AGCAGG---------TACCCTTCAGGAACAAA	-9
	AG-------------TACCCTCCAGGAACAAA	-13
	------------TAGTACCCTTCAGGAACAAA	-12
	---------------TACCCTTCAGGAACAAA	-27
	----------------------------CAAA	-31
	----------------------------CAAA	-44

N: 17	TatRev	Size (bp)
	CAAGGCAGTCAGACTCATCAAGTTTCTCTATCAAA	
2 ×	CAAGGCAGTCAGACTCATCAAGTTTCTCTATCAAA	WT
	CAAGGCAGTCAGAC--ATCAAGTTTCTCTATCAAA	-2
	CAAGGCAGTC----GGATCAAGTTTCTCTATCAAA	-4 +2
2 ×	CCAGGCAGTC------ATCAAGTTTCTCTATCAAA	-6
	CAAGGCA-------TCATCAAGTTTCTCTATCAAA	-7
	CAAGGCAG--------ATCAAGTTTCTCTATCAAA	-8
	CA------------TCATCAAGTTTCTCTATCAAA	-12
	CA-----------------TCAAGTTTCTCTATCAAA	-15
	CAAGG----------------------AGGCCAAA	-22 +4
	-------------------TCAAGTTTCTCTATCAAA	-19
	-------------------TCAAGTTTCTCTATCAAA	-21
	----------------------------TATCAAA	-28
	----------------------TTTCTCTATCAAA	-29
	--------------------------GGTATCAAA	-35 +2
	-----------------------------------	-178

Figure S3. **Sequence analysis of HIV proviral DNA in Gag1-Tat1, Gag1-Tat2 and Gag1-TatRev crRNA-protected SupT1 cells, related to Figure 5.** Cellular DNA was isolated at 30 and 60 days after HIV infection. The crRNA-targeted region was amplified by PCR and TA-cloned. Multiple TA clones were sequenced. Sequences were aligned to the wild-type HIV LAI isolate, which is the wild-type (WT) shown at the top. The crRNA targets are underlined and the PAM is marked in blue. Deletions were labelled with dashes. Substitutions are highlighted in red. Unedited WT sequences were marked in grey background. In front of the sequences, we indicated those that were detected multiple times.

Table S1. Days when Cas12a resistant HIV replicated in CRISPR-Cas12a transduced cells

Culture	1	2	3	4	5	6
Gag1+LTR1	7 days	7 days	7 days	7 days	7 days	7 days
Gag1+Tat2	13 days	14 days	-	-	-	-
Gag1+TatRev	14 days	9 days	-	-	-	-
Tat1+TatRev	23 days	-	-	-	-	-
Tat2+TatRev	20 days	19 days	29 days	20 days	19 days	-

Table S2. Primers used for PCR and sequencing

Primer name		Oligonucleotide Sequence (5'-3')
a :	5LTR_forward	CAAGATATCCTTGATCTGTGG
b :	5LTR_reverse	TTCTGATGTTTCTAACAGGC
c :	3LTR_forward	GCTTGTGCCTGGCTAGAAGC
d :	3LTR_reverse	CTGGAAAGTCCCCAGCGGAA
e :	crGag1_forward	ACCTAGAACTTTAAATGCATGG
f :	crGag1_reverse	CGGTCTACATAGTCTCTAAAGG
g :	crTatRev_forward	AACTTATGGGGATACTTGGG
h :	crTarRev_reverse	CTATGATTACTATGGACCACACA
	T7	TAATACGACTCACTATAGGG
	M13RP	CAGGAAACAGCTATGAC
